# *Eimeria* spp. and *Tyzzeria perniciosa* Allen, 1936 (Apicomplexa: Eimeriidae) from a Pacific black duck, *Anas superciliosa* Gmelin (Aves: Anseriformes), in western Australia

**DOI:** 10.1016/j.crpvbd.2022.100075

**Published:** 2022-01-07

**Authors:** Bruno P. Berto, Belinda Brice, Gwyneth Thomas, Aileen Elloit, Alireza Zahedi, Rongchang Yang

**Affiliations:** a Departamento de Biologia Animal, Instituto de Ciências Biológicas e da Saúde, Universidade Federal Rural do Rio de Janeiro, BR-465 km 7, Seropédica, RJ, 23897-000, Brazil; b Kanyana Wildlife Rehabilitation Centre, 120 Gilchrist Road, Lesmurdie, WA 6076, Australia; c College of Science, Health, Engineering and Education, Murdoch University, Harry Perkins Building, Perth, WA 6150, Australia; d The Centre of Biosecurity and One Health, Harry Butler Institute, Murdoch University, Perth, WA 6150, Australia; e Australian National Phenome Centre, Health Futures Institute, Murdoch University, Harry Perkins Building, Perth, WA 6150, Australia

**Keywords:** Coccidia, *Eimeria*, *Tyzzeria*, Pacific black duck, *Anas superciliosa*, 18S rRNA gene, *cox*1 gene

## Abstract

Four species of the Eimeriidae, *Eimeria anatis* Scholtyseck, 1955, *Eimeria aythyae* Farr, 1965, *Eimeria krylovi* Svanbaev & Rakhmatullina, 1967 and *Tyzzeria perniciosa* Allen, 1936, were morphologically identified from oöcysts recovered from a Pacific black duck, *Anas superciliosa* Gmelin. Additionally, genotypic characterization of *E. anatis* is provided *via* sequencing of the mitochondrial cytochrome *c* oxidase subunit 1 (*cox*1) and the small subunit ribosomal RNA (18S) genes. The four species are redescribed, providing additional morphological details. The validity of genera and coccidian species parasitizing birds of the order Anseriformes such as *Wenyonella* Hoare, 1933 and some *Tyzzeria* spp. are discussed. Molecular phylogenetic analyses for the *cox*1 and 18S rRNA genes resulted in monophylies of *Eimeria* spp. from Anseriformes which included the sequences obtained from *E. anatis* oöcysts.

## Highlights

• *Eimeria anatis*, *Eimeria aythyae*, *Eimeria krylovi*, *Tyzzeria perniciosa* are identified from a new host, *Anas superciliosa.*
• Coccidia are redrawn and redescribed to offer better characterization of the morphological features.
• Genotypic characterization *via* sequencing of mitochondrial and ribosomal genes is provided for *E. anatis.*
• Taxonomic validity of coccidia parasitic in Anseriformes, such as *Wenyonella* and some *Tyzzeria* spp. is discussed.

## Graphical abstract

**Figure:**
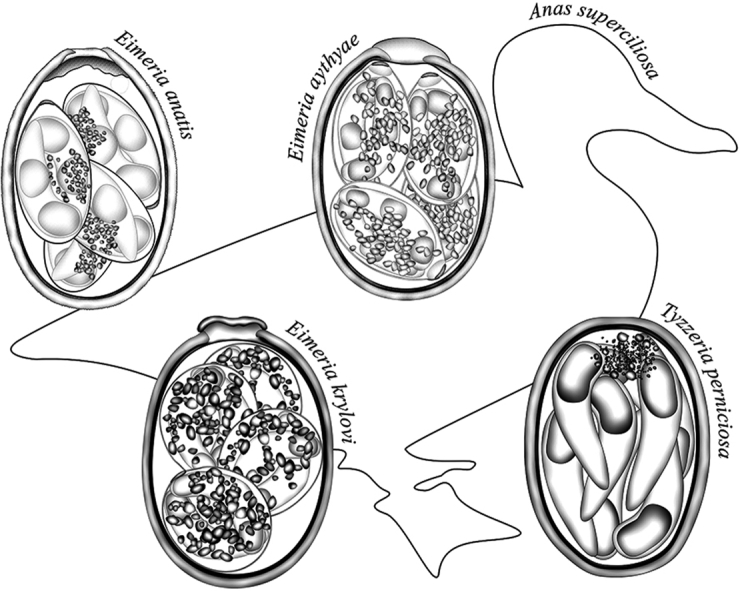

## 1 Introduction

The Pacific black duck *Anas supercilios*a Gmelin (Anseriformes: Anatidae) is a dabbling duck commonly seen in waterways, swamps, streams and ponds in Australia, New Zealand, Indonesia through to Papua New Guinea, Polynesia, the islands of the West Pacific and the sub-Antarctic islands (Pizzey and Knight, 2007).

Both domestic and wild ducks are commonly infected with gastrointestinal parasites including coccidia, which are obligate intracellular protozoans of the Apicomplexa (Gajadhar et al., 1983). Species of *Eimeria* Schneider, 1875 (Eimeriidae) are the most common coccidia found in birds, including ducks, with mixed infections being common (Leibovitz, 1968). *Eimeria anatis* Scholtyseck, 1955 infects the mallard *Anas platyrhynchos* (L.) (Duszynski et al., 2001) and *Eimeria aythyae* Farr, 1965 infects the lesser scaup *Aythya affinis* (Eyton) (Gajadhar et al., 1983; Duszynski et al., 2001). Windingstad et al. (1980) reported recurring epizootic infection in *A. affinis* resulting from infection with *E. aythyae*. The host range of *Eimeria krylovi* Svanbaev & Rakhmatullina, 1967 includes the green-winged teal *Anas carolinensis* Gmelin, the northern shoveler *Spatula clypeata* (L.), the European wigeon *Mareca penelope* (L.), the gadwall *Mareca strepera* (L.) and the garganey *Spatula querquedula* (L.) (Svanbaev & Rakhmatullina, 1967).

Coccidia of the genus *Tyzzeria* Allen, 1936, have also been described predominantly from ducks (Gajadhar et al., 1983). This genus is made up of coccidia whose oöcysts lack sporocysts (Duszynski et al., 1998). Cole & Friend (1999) reported that *Tyzzeria* spp. were less commonly seen in ducks than *Eimeria* spp. *Tyzzeria perniciosa* Allen, 1936 is an important pathogenic coccidian in ducks and is especially pathogenic for ducklings (Baker, 2007). Reported duck hosts of *T. perniciosa* are the northern pintail *Anas acuta* L., the lesser scaup *A. affinis*, the common shelduck *Tadorna tadorna* (L.), the tufted duck *Aythya fuligula* (L.), the mallard *A. platyrhynchos* and the white-headed duck *Oxyura leucocephala* (Scopoli) (Duszynski et al., 1998). A study from China reported outbreaks of coccidiosis due to *T. perniciosa* and *Wenyonella philiplevinei* Leibovitz, 1968, amongst farmed ducklings (Peiyun et al., 1982). A study in Iraq detected *E. anatis* in 17% and *T. perniciosa* in 11% of domesticated ducks screened (*n* = 80) (Abdullah, 2010) while another study on domestic ducks in Iran found a variety of protozoan parasites including *Cryptosporidium* spp., *Tyzzeria* spp., *W. philiplevinei*, *Isospora mandari* Bhatia, Chauhan, Arora & Agrawal, 1971 as well as other coccidian species (Larki et al., 2018).

The coccidia infecting ducks are similar in size and have very similar morphologies. This makes identification difficult using morphology alone (Leibovitz, 1968; Gajadhar et al., 1983). Those coccidia infecting wild ducks have not been well studied. In this study, we morphologically identified *E. anatis*, *E. aythyae*, *E. krylovi* and *T. perniciosa* from a Pacific black duck. Additionally, we provided genotypic characterization *via* sequencing of the mitochondrial cytochrome *c* oxidase subunit 1 (*cox*1) and the small subunit ribosomal RNA (18S) genes for *E. anatis*.

## 2 Materials and methods

### 2.1 Sample collection and examination

A wild, juvenile Pacific black duck was admitted to the Kanyana Wildlife Rehabilitation Centre (KWRC), Perth, Australia, in January 2021, after it was struck by a motor vehicle. Physical examination on admission revealed no external injuries; however, the duck was extremely quiet, reluctant to walk and was leaning to one side. It had a body condition score of 2/5. The duck was given supportive treatment of fluids and pain relief medication before being sent to a veterinarian for further assessment. A preliminary diagnosis of concussion and possible internal injuries was made. A faecal sample was collected on admission to KWRC. Initial direct light microscopy revealed a heavy, mixed parasitic load including large numbers of unsporulated coccidian oöcysts of various sizes as well as trophozoites of *Trichomonas* Donné, 1836, eggs of *Capillaria* Zeder, 1800 and tapeworm eggs. The duck was treated for the worm infection with praziquantel and moxidectin (20 mg/kg and 1 mg/kg of each ingredient respectively), *per os* (PO), once daily (OD), which was repeated after 14 days. The coccidia were treated with toltrazuril (15 mg/kg, PO, OD) for three consecutive days and then again 7 days later. Metronidazole (50 mg/kg, PO, OD) was given for 7 days for the *Trichomonas* infection. The duck made a full recovery and was released near the found location 4 weeks later.

A portion of faeces was placed in 2% (w/v) K_2_Cr_2_O_7_, mixed well and placed in a refrigerator, until transport to Murdoch University (within 48 h) for further investigation. On arrival at the Murdoch University laboratory, the faecal solution was poured into a Petri dish (to a depth of less than 1 cm). The Petri dish was stored in a dark environment and kept at room temperature (22 °C), to facilitate sporulation. The sample was checked daily for oöcyst sporulation using an Olympus DP71 digital microimaging camera. Sporulated oöcysts were observed using the 100× oil immersion objective. Images were taken using Nomarski contrast with a 100× oil immersion objective. Line drawings were edited using two software applications of CorelDRAW® (Corel Draw Graphics Suite, Version, 2020; Corel Corporation, Canada), i.e. Corel DRAW and Corel PHOTO-PAINT. All measurements are in micrometres and are given as the range followed by the mean in parentheses.

### 2.2 Oöcyst isolation, DNA extraction, PCR amplification, sequencing and phylogenetic analyses

Five morphologically similar oöcysts were isolated for a bulk DNA extraction with the method described by Yang et al. (2015). The DNA extraction, PCR amplification of the 18S rRNA and *cox*1 genes and sequencing were conducted according to the protocols described by Yang et al. (2013, 2016).

Phylogenetic trees were constructed for *E. anatis* using partial 18S rDNA and partial *cox*1 sequences aligned with additional species/isolates from GenBank using ClustalW (http://www.phylogeny.fr/one_task.cgi?task_type=clustalw). Distance analyses and phylogenies were conducted using MEGA X (Kumar et al., 2018) as described in detail by Yang et al. (2021) with the most appropriate nucleotide substitution models (TN93 + G + I for 18S and TN93 + G for the *cox*1 gene). Bootstrap support was estimated from 1000 pseudoreplicates.

## 3 Results

Based on the morphological analysis of the coccidian oöcysts in the faecal sample, four species were identified: *E. anatis*, *E. aythyae*, *E. krylovi* and *T. perniciosa*. The newly collected material is described below.

### 3.1 *Eimeria anatis* Scholtyseck, 1955

**Fig. 1:**
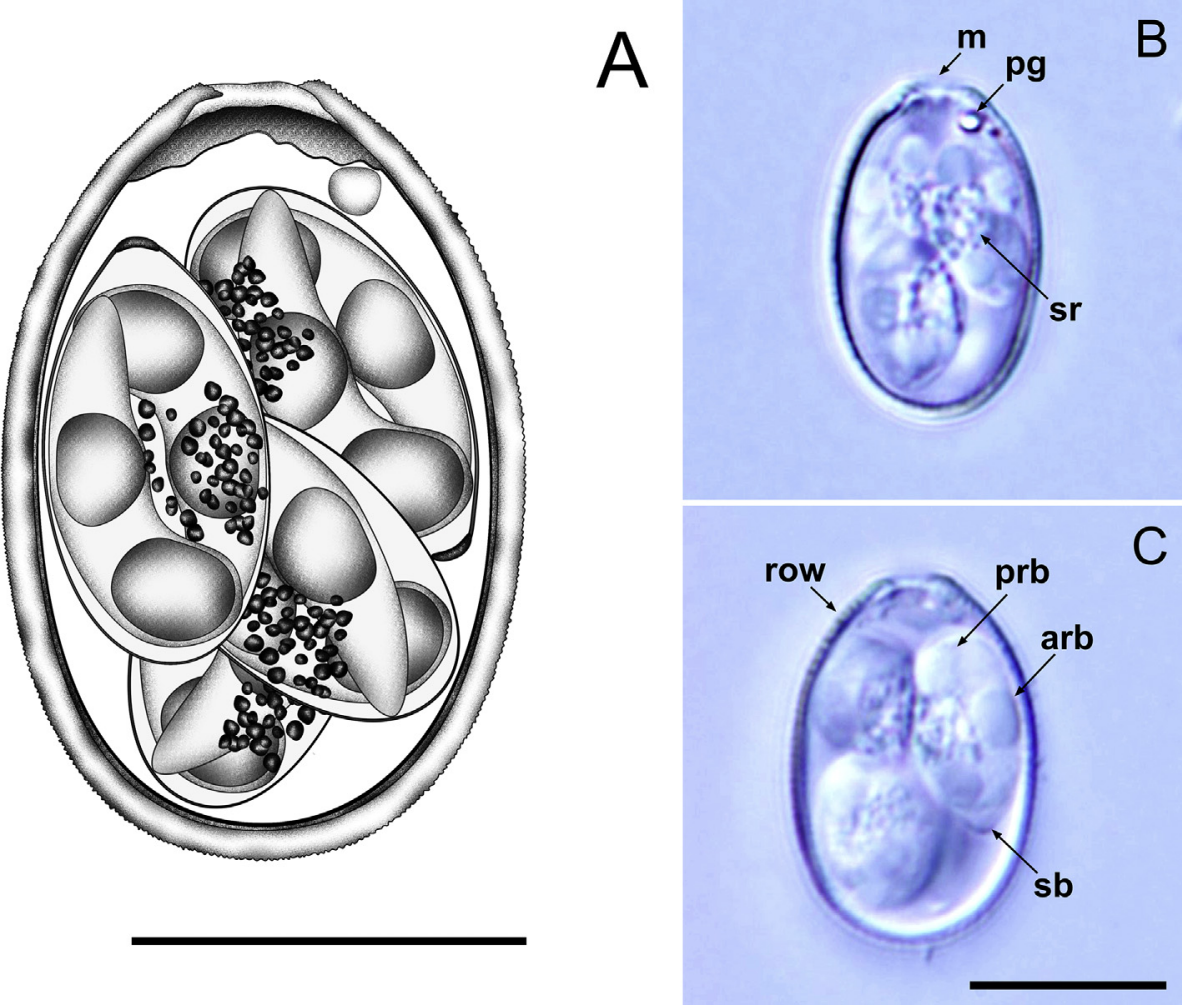
Composite line drawing (**A**) and photomicrographs (**B**, **C**) of sporulated oöcysts of *Eimeria anatis* from the Pacific black duck *Anas superciliosa*. Note the anterior (arb) and posterior (prb) refractile bodies; micropyle (m); polar granule (pg); rough oöcyst wall (row); Stieda body (sb); and sporocyst residuum (sr). *Scale-bars*: 10 μm.

[Description based on 20 oöcysts and 40 sporocysts; Fig. 1.] Oöcysts elongate-ovoidal, 17–19 × 11–13 (17.6 × 11.9); length/width (L/W) ratio 1.4–1.6 (1.5). Oöcyst wall bi-layered, 0.9–1.3 (1.0) thick; outer layer smooth to slightly rough, *c.*2/3 of total thickness. Micropyle cap absent. Micropyle present, generally with invagination of inner layer. Oöcyst residuum absent, but 1–2 polar granules present. Sporocysts 4, ellipsoidal, 7–9 × 5–6 (7.9 × 5.9); L/W ratio 1.3–1.4 (1.3). Stieda body present, flattened; sub-Stieda absent or indiscernible; para-Stieda body absent. Sporocyst residuum present, composed of small, randomly dispersed granules. Sporozoites 2, with robust anterior and posterior refractile bodies and indiscernible nucleus.

### 3.2 *Eimeria aythyae* Farr, 1965

**Fig. 2:**
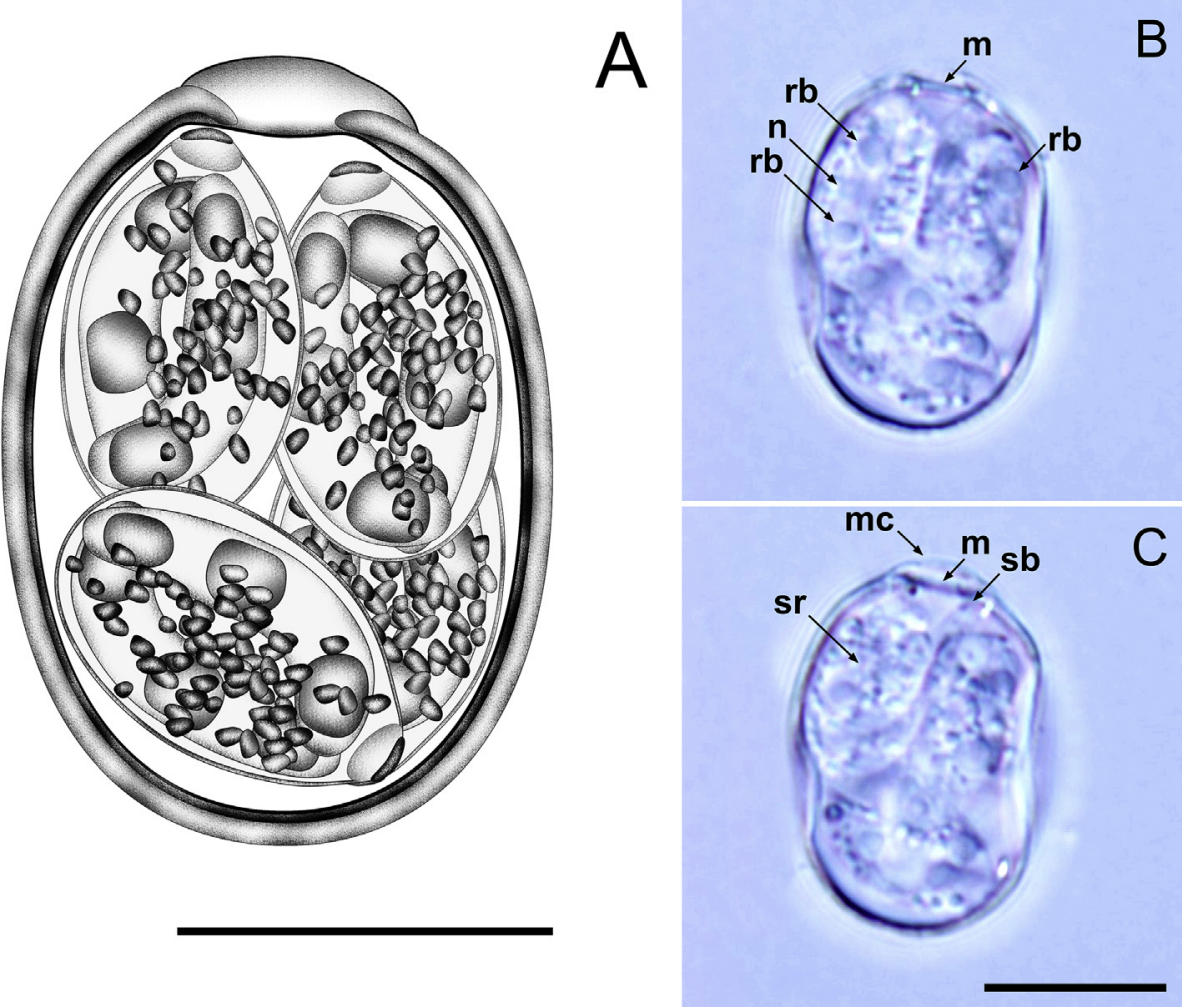
Composite line drawing (**A**) and photomicrographs (**B**, **C**) of sporulated oöcysts of *Eimeria aythyae* from the Pacific black duck *Anas superciliosa*. Note the micropyle (m); micropyle cap (mc); nucleus (n); Stieda body (sb); sporocyst residuum (sr); and refractile body (rb). *Scale-bars*: 10 μm.

[Description based on 20 oöcysts and 40 sporocysts; Fig. 2.] Oöcysts ellipsoidal, 20–23 × 14–16 (21.3 × 15.2); L/W ratio 1.3–1.5 (1.4). Oöcyst wall bi-layered, 1.0–1.4 (1.2) thick; outer layer smooth, *c.*2/3 of total thickness. Micropyle cap present as a translucent, delicate, curved protrusion. Micropyle present with no invagination of inner layer. Oöcyst residuum and polar granule absent. Sporocysts 4, ellipsoidal, 9–11 × 7–8 (10.5 × 7.7); L/W ratio 1.2–1.4 (1.4). Stieda body present, flattened; sub-Stieda present, but delicate or indiscernible in some sporocysts; para-Stieda body absent. Sporocyst residuum present, composed of large, randomly dispersed granules. Sporozoites 2, with robust anterior and posterior refractile bodies and centrally located nucleus.

### 3.3 *Eimeria krylovi* Svanbaev & Rakhmatullina, 1967

**Fig. 3:**
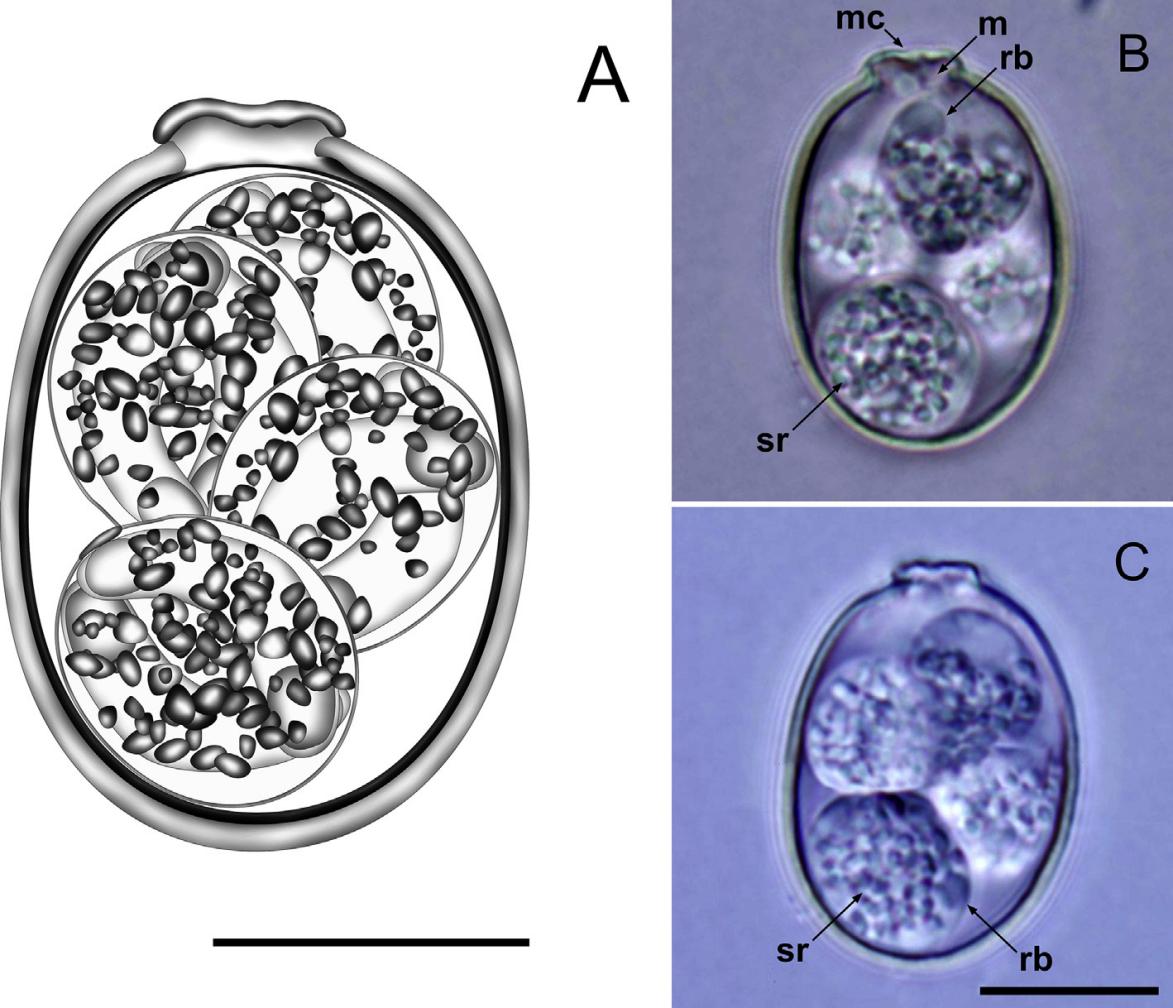
Composite line drawing (**A**) and photomicrographs (**B**, **C**) of sporulated oöcysts of *Eimeria krylovi* from the Pacific black duck *Anas superciliosa*. Note the micropyle (m); micropyle cap (mc); sporocyst residuum (sr); and refractile body (rb). *Scale-bars*: 10 μm.

[Description based on 25 oöcysts and 50 sporocysts; Fig. 3.] Oöcysts ellipsoidal, 20–23 × 16–17 (21.7 × 16.1); L/W ratio 1.3–1.4 (1.3). Oöcyst wall bi-layered, 1.0–1.4 (1.2) thick; outer layer smooth, *c*.2/3 of total thickness. Micropyle cap present as a dense cover. Micropyle present with no invagination of inner layer. Oöcyst residuum and polar granule absent. Sporocysts 4, subspheroidal to ellipsoidal, 8–10 × 7–8 (8.8 × 7.7); L/W ratio 1.1–1.2 (1.1). Stieda body flattened, barely or not discernible; sub-Stieda absent; para-Stieda body absent. Sporocyst residuum present, composed of many large and dense granules which are widely diffused within the sporocyst. Sporozoites 2, with anterior and posterior refractile bodies and indiscernible nucleus.

### 3.4 *Tyzzeria perniciosa* Allen, 1936

**Fig. 4:**
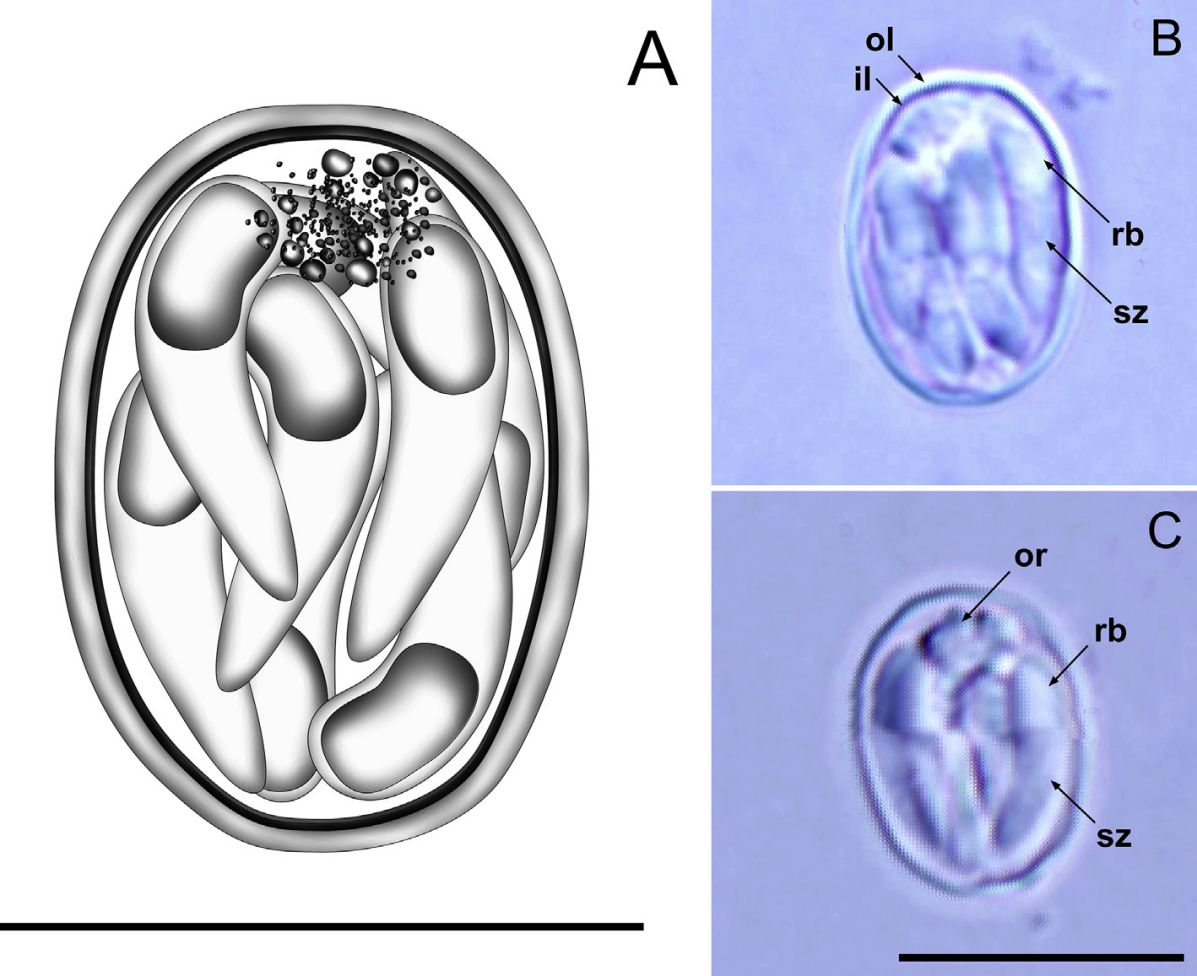
Composite line drawing (**A**) and photomicrographs (**B**, **C**) of sporulated oöcysts of *Tyzzeria perniciosa* from the Pacific black duck *Anas superciliosa*. Note the inner (il) and outer (ol) layers of the oöcyst wall; oöcyst residuum (or); refractile body (rb); and sporozoite (sz). *Scale-bars*: 10 μm.

[Description based on 25 oöcysts; Fig. 4.] Oöcysts ellipsoidal, 10–11 × 7–8 (10.7 × 7.4); L/W ratio 1.4–1.5 (1.4). Oöcyst wall bi-layered, 0.4–0.7 (0.6) thick; outer layer smooth, *c*.2/3 of total wall thickness. Oöcyst residuum present as granules of different sizes usually clustered at one end of oöcyst, measuring *c.*2.5. Sporozoites 8, curved and tapered at anterior end, 6–8 × 1–2 (6.9 × 1.8), with robust, prominent posterior refractile body and without discernible nucleus.

### 3.5 Molecular identification

PCR amplification for the 18S rRNA and *cox*1 genes from oöcyst DNA of the four coccidian species were conducted; unfortunately, PCR amplicons were successfully obtained only for *E. anatis* oöcysts.

#### 3.5.1 Phylogenetic analyses of the 18S rRNA gene

**Fig. 5:**
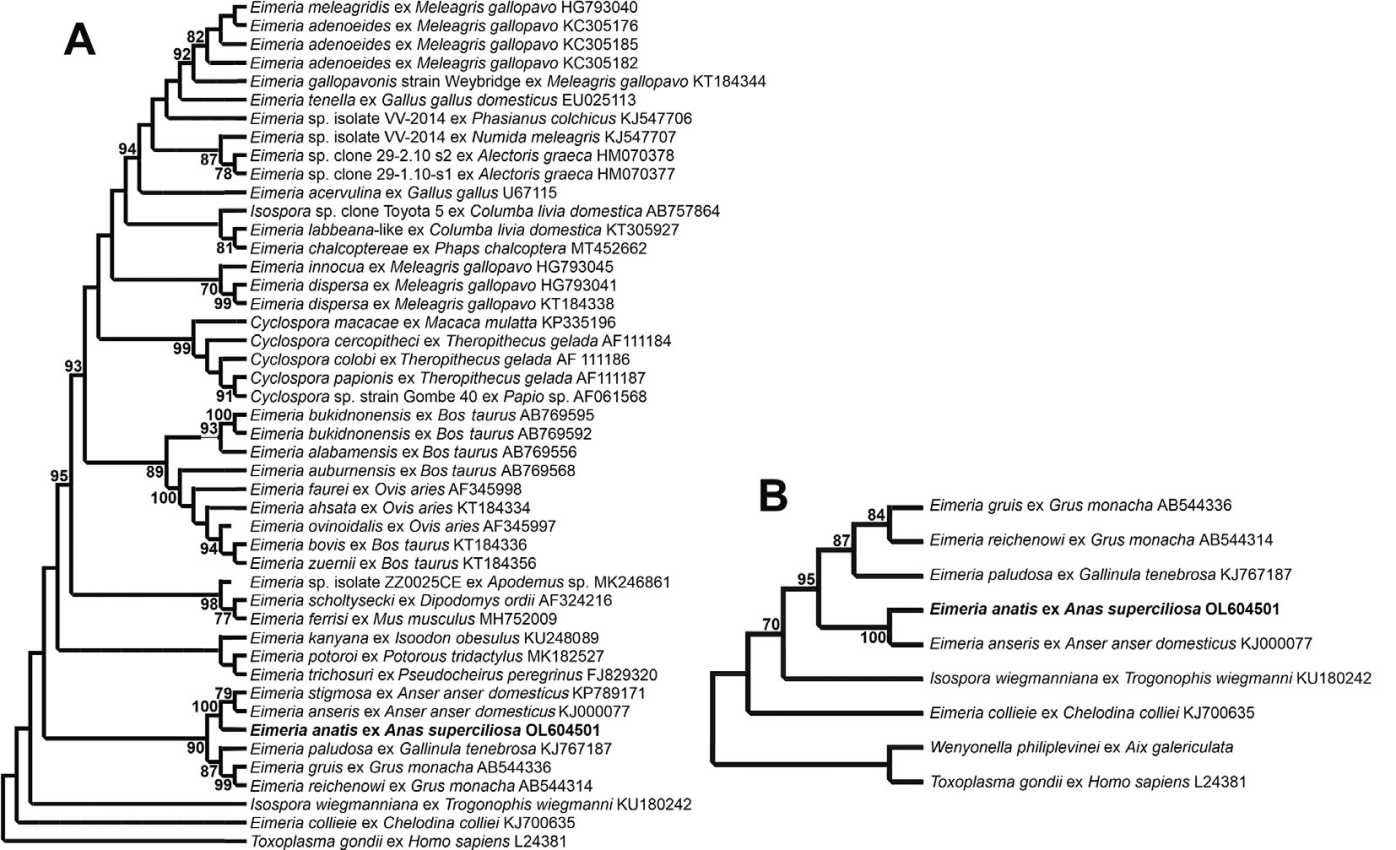
Evolutionary relationships of *Eimeria anatis* inferred by maximum likelihood analysis (ML) of 18S rDNA sequences (**A**, alignment length 1209 bp; **B**, alignment length 424 bp). Percentage support (> 70%) from 1000 pseudoreplicates from the ML analysis is indicated at the nodes.

A 1209 bp 18S rDNA sequence with clean sequencing chromatography was obtained from the five morphological similar oöcysts of *E. anatis* isolated from the faecal samples of *Anas superciliosa*; this was aligned with 37 sequences for *Eimeria* spp., 5 for *Cyclospora* spp. and 2 for *Isospora* spp. based on the NCBI BLAST similarities. The alignment covered all available *Eimeria* spp. sequences. A 18S rRNA gene sequence (GenBank: L24381) of *Toxoplasma gondii* (Nicolle & Manceaux, 1908) was used as the outgroup. *Eimeria anatis* shared 97.6% and 96.6% similarity with *Eimeria stigmosa* Klimes, 1963 (GenBank: KP789171) and *Eimeria anseris* Kotlan, 1932 (GenBank: KJ000077), respectively, both of which were obtained from *Anser anser* (L.) in China (sequences published in GenBank only). *Eimeria anatis* also shared a genetic similarity of 94.6% with both *Eimeria gruis* Yakimoff & Matschoulsky, 1935 (GenBank: AB544336) and *Eimeria reichenowi* Yakimoff & Matschoulsky, 1935 (GenBank: AB544314), both identified from *Grus monacha* Temminck in Japan and reported by the same group (Honma et al., 2011). In addition, *E. anatis* shared 93.0% similarity with *Eimeria paludosa* (Leger & Hesse, 1922) (GenBank: KJ767187) from *Gallinula tenebrosa* Gould in Western Australia (Yang et al., 2014). As shown in Fig. 5A, *E. anatis* was placed in a separate strongly supported clade with *E. stigmosa* and *E. anseris*, closely associated with a sister clade composed of *E. gruis*, *E. reichenowi* and *E. paludosa*.

*Eimeria anatis* is often related to the coccidian species *W. philiplevinei*; however, there is no 18S DNA sequence from *W. philiplevinei* available, only a 422-bp 18S sequence presented in the paper by Wu et al. (2013). The 18S sub-tree generated from a shortened alignment including both *E. anatis* and *W. philiplevinei* showed that *E. anatis* belongs to the same clade as that of the 18S phylogenetic tree based on the long alignment (Fig. 5A), whereas *W. philiplevinei* was positioned close to *T. gondii*, outside of the *Eimeria* spp. clades (Fig. 5B). The genetic similarity between *E. anatis* and *W. philiplevinei* was 86.5%.

#### 3.5.2 Phylogenetic analyses of the *cox*1 gene

**Fig. 6:**
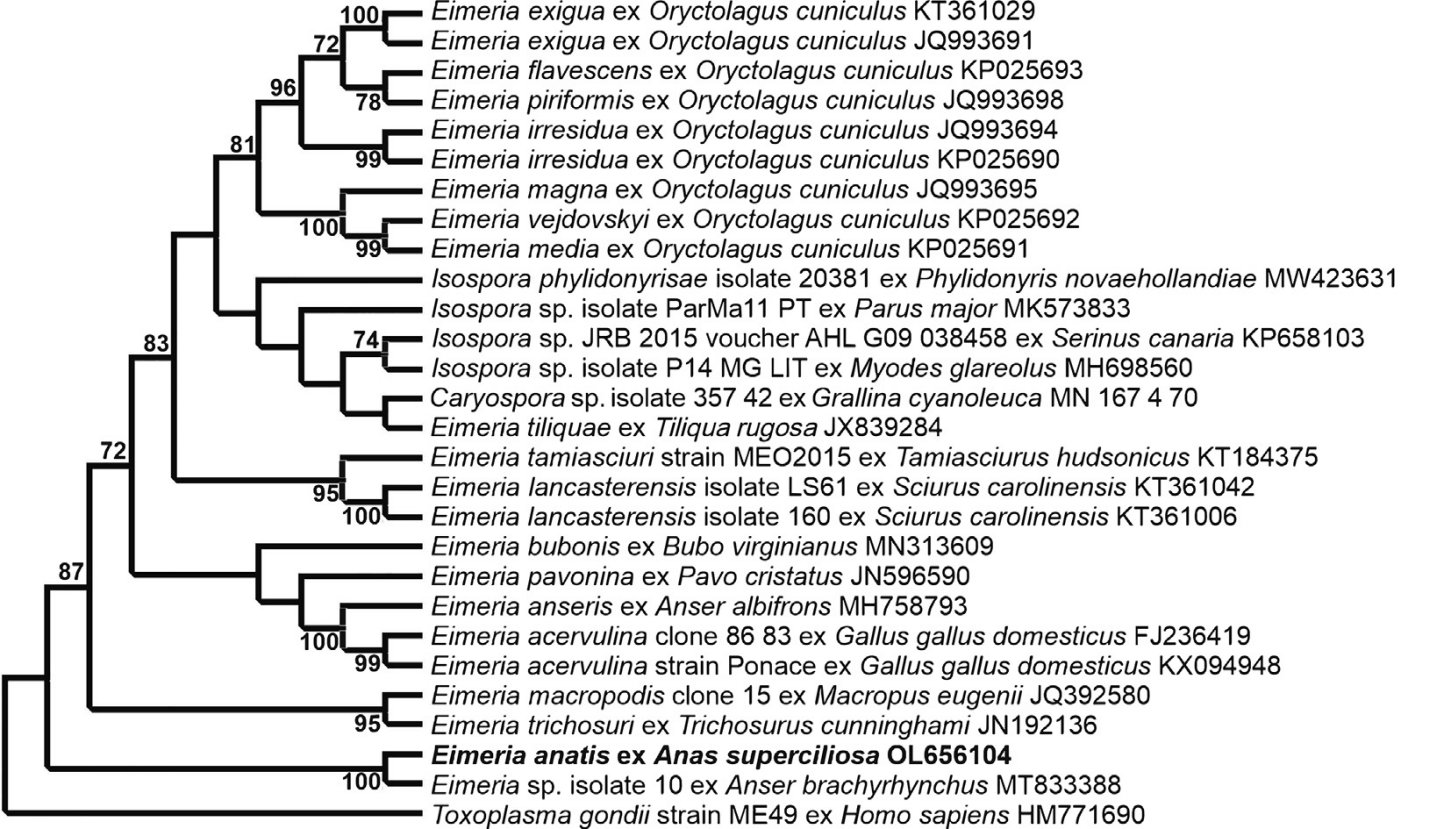
Evolutionary relationships of *Eimeria anatis* inferred by maximum likelihood analysis (ML) of partial *cox*1 gene sequences (650 bp). Percentage support (> 70%) from 1000 pseudoreplicates from the ML analysis is indicated at the nodes.

The *cox*1 gene was amplified from *E. anatis* oöcyst DNA and a 650-bp sequence was successfully obtained and aligned with 21 sequences for *Eimeria* spp. from different animal species, 4 for *Isospora* spp. and one for *Caryospora* sp. All *cox*1 reference sequences were selected based on the NCBI BLAST similarities and covered all *Eimeria* spp. in the database. A sequence for *T. gondii* (GenBank: HM771690) was used as the outgroup. *Eimeria anatis* showed the highest genetic similarity (91.9%) with an unnamed *Eimeria* sp. isolated from the pink-footed goose *Anser brachyrhynchus* Baillon (GenBank: MT833388) (Myšková et al., 2021) and grouped with this *Eimeria* sp. in the same clade in the phylogenetic tree (Fig. 6).

## 4 Discussion

*Eimeria anatis*, *E. aythyae*, *E. krylovi* and *T. perniciosa* have all been previously reported to infect ducks. The Pacific black duck in this study was found to be infected with all three of these *Eimeria* spp. simultaneously as well as *T. perniciosa*. This is not unusual as co-infections with *Eimeria* spp. are regularly observed in birds.

The oöcysts of the three *Eimeria* spp. identified in this study were morphologically compatible with their respective original descriptions (Table 1, Table 2). However, it is noteworthy that in the present study some adjustments for some characteristic features were added to the descriptions of these species. For example, a Stieda body and a sub-Stieda body were observed in *E. anatis* and *E. aythyae*, respectively, which were not identified in the original descriptions (Scholtyseck, 1955; Farr, 1965) or in later reports (Gajadhar et al., 1983). *Eimeria krylovi* was not originally described with a Stieda body, and indeed this structure was hardly observed and photomicrographed in this study, being reported here as “barely or not discernible”. In this context, it is important to highlight that a Stieda body is a synapomorphic characteristic of the family Eimeriidae, and its lack indicates a change in the identification to another genus and/or family, such as the genera *Choleoeimeria* Paperna & Landsberg, 1989, *Acroemeria* Paperna & Landsberg, 1989, *Goussia* Labbé, 1896, etc., which were originally described within *Eimeria*, but due to the absence of a Stieda body, among other aspects, classified in other genera (Jirků et al., 2009). In the present study, these three *Eimeria* spp. were redrawn and redescribed to offer better characterization of features such as micropyle, micropyle cap, Stieda body, sub-Stieda body, sporocyst residuum and refractile bodies and sporozoite nucleus, aiming to facilitate and enable reliable identification of these species in further studies.

**Table 1:** Comparative morphology of the oöcysts of *Eimeria* spp. recorded from ducks (Anseriformes: Anatidae: Anatinae)

Species	Host	Shape	Size (μm)^a^	Shape index	Polar granule	Wall (μm)^a^	Micropyle (μm)^a^	Reference
*Eimeria abramovi* Svanbaev & Rakhmatullina, 1967	*Anas platyrhynchos* (L.)	Ovoidal to ellipsoidal	21–22 × 16–17	–	Absent	Smooth, (1.4) thick	Present, (2.4) wide, with micropyle cap	Svanbaev & Rakhmatullina, 1967
*Eimeria anatis* Scholtyseck, 1955	*A. platyrhynchos*	Ovoidal	14–19 × 11–16 (16.8 × 14.1)	–	–	Smooth, 0.7–1.0 thick	Present, closed by a plug-like mass	Scholtyseck (1955)
	*Anas superciliosa* Gmelin	Elongate-ovoidal	17–19 × 11–13 (17.6 × 11.9)	1.4–1.6 (1.5)	Present, 1–2	Smooth to slightly rough, 0.9–1.3 (1.0) thick	Present, with an invagination of the inner layer, without micropyle cap	Present study
*Eimeria aythyae* Farr, 1965	*Aythya affinis* (Eyton)	Broadly ellipsoidal to a round-bottomed urn with shoulder	15–24 × 10–18 (20.1 × 15.5)	–	Absent	Smooth or lightly sculptured, 0.6–0.8 thick	Present, (3.6) wide	Farr (1965)
	*A. superciliosa*	Ellipsoidal	20–23 × 14–16 (21.3 × 15.2)	1.3–1.5 (1.4)	Absent	Smooth, 1.0–1.4 (1.2) thick	Present, without invagination of the inner layer; micropyle cap as a translucent and delicate curved protrusion	Present study
*Eimeria battakhi* Dubey & Pande, 1963	*A. platyrhynchos*	Subspherical to ovoidal	19–24 × 16–21 (21.0 × 18.0)	1.1–1.2	Present, 1	Smooth, 1.0–2.0 thick	Absent	Dubey & Pande (1963)
*Eimeria boschadis* Waldén, 1961	*A. platyrhynchos*	Bottle-shaped	18–27 × 12–13 (23.9 × 12.7)	–	–	Finely granulated	Present, 2–3 wide	Waldén (1961)
*Eimeria bucephalae* Christiansen & Madsen, 1948	*Bucephala clangula* (L.)	Elongate-ovoidal	25–39 × 13–20 (30.3 × 15.6)	–	–	–	Present, narrow	Christiansen & Madsen (1948)
*Eimeria danailovi* Grafner, Graubmann & Betke, 1965	*Anas platyrhynchos*	Ovoidal	19–23 × 11–15 (18.7 × 12.5)	–	–	0.6–1.0 thick	Present	Gräfner et al. (1965)
*Eimeria koganae* Svanbaev & Rakhmatullina, 1967	*Spatula querquedula* (L.)	Ovoidal or ellipsoidal	21–25 × 13–21 (21.5 × 16.1)	–	Absent	0.8–1.0 thick	Present, (5.0) wide	Svanbaev & Rakhmatullina, 1967
*Eimeria krylovi* Svanbaev & Rakhmatullina, 1967	*Anas carolinensis* Gmelin; *Spatula clypeata* (L.); *S. querquedula*; *Mareca penelope* (L.); *Mareca strepera* (L.)	Subspherical	15–21 × 13–17	–	Present, 1	Smooth, 1.2 thick	Present at the flattened end, 4.0–6.0 wide, covered by a (4.0) wide and (2.0) high micropyle cap	Svanbaev & Rakhmatullina, 1967
	*Anas superciliosa* Gmelin	Ellipsoidal	20–23 × 16–17 (21.7 × 16.1)	1.3–1.4 (1.3)	Absent	Smooth, 1.0–1.4 (1.2) thick	Present, without invagination of the inner layer, with micropyle cap as a dense cover	Present study
*Eimeria nyroca* Svanbaev & Rakhmatullina, 1967	*Aythya nyroca* (Güldenstädt)	Ovoidal	21–40 × 17–19 (25.4 × 17.7)	–	Absent	Smooth, 1.0–2.0 thick	Present, surrounded by collars, 4.0–6.0 wide, with 2.0–3.0 high micropyle cap	Svanbaev & Rakhmatullina, 1967
*Eimeria saitamae* Inoue, 1967	*A. platyrhynchos*	Ovoidal	17–21 × 13–15 (18.6 × 13.2)	–	Present, 1	Smooth, 0.7–0.8 thick	Present	Inoue (1967)
*Eimeria schachdagica* Musaev, Surkova, Jelchiev & Alieva, 1966	*A. platyrhynchos*	Ovoidal	16–26 × 12–20 (24.0 × 17.7)	–	Present, refractile granules	Smooth, 1.6–2.0 thick	Absent	Musaev et al. (1966)
*Eimeria somateriae* Christiansen, 1952	*Clangula hyemalis* (L.)	Bottle-shaped, asymmetric	21–41 × 11–19 (31.9 × 13.9)	–	–	Smooth, thin, colorless	Present	Christiansen (1952)

*Note*: *E. boschadis* and *E. somateriae* are kidney parasites, while the remaining species are intestinal parasites.

a Range (Mean).

**Table 2:** Comparative morphology of the sporocysts of *Eimeria* spp. recorded from ducks (Anseriformes: Anatidae: Anatinae)

Species	Host	Shape	Size (μm)^a^	Shape index	Stieda body	Sub-Stieda body	Sporocyst residuum	Reference
*Eimeria abramovi* Svanbaev & Rakhmatullina, 1967	*Anas platyrhynchos* (L.)	–	7–9 × 5	–	–	–	Present, small granules	Svanbaev & Rakhmatullina, 1967
*Eimeria anatis* Scholtyseck, 1955	*A. platyrhynchos*	Ovoidal	–	–	–	–	Present, few central granules	Scholtyseck (1955)
	*Anas superciliosa* Gmelin	Ellipsoidal	7–9 × 5–6 (7.9 × 5.9)	1.3–1.4 (1.3)	Present, flattened	Absent or indiscernible	Present, small granules randomly dispersed	Present study
*Eimeria aythyae* Farr, 1965	*Aythya affinis* (Eyton)	–	–	–	Present, small	–	Present, compact residual mass	Farr (1965)
	*A. superciliosa*	Ellipsoidal	9–11 × 7–8 (10.5 × 7.7)	1.2–1.4 (1.4)	Present, flattened	Present, delicate or indiscernible	Present, large granules randomly dispersed	Present study
*Eimeria battakhi* Dubey & Pande, 1963	*A. platyrhynchos*	Ovoidal	11–13 × 6–8	–	Present, small	–	Present	Dubey & Pande (1963)
*Eimeria boschadis* Waldén, 1961	*A. platyrhynchos*	–	–	–	–	–	–	Waldén (1961)
*Eimeria bucephalae* Christiansen & Madsen, 1948	*Bucephala clangula* (L.)	–	–	–	–	–	–	Christiansen & Madsen (1948)
*Eimeria danailovi* Grafner, Graubmann & Betke, 1965	*Anas platyrhynchos* (L.)	Ovoidal	–	–	–	–	Present	Gräfner et al. (1965)
*Eimeria koganae* Svanbaev & Rakhmatullina, 1967	*Spatula querquedula* (L.)	Subspherical to ovoidal	9–11 × 8–10	–	–	–	Present, clear globules irregularly spaced	Svanbaev & Rakhmatullina, 1967
*Eimeria krylovi* Svanbaev & Rakhmatullina, 1967	*Anas carolinensis* Gmelin; *Spatula clypeata* (L.); *S. querquedula*; *Mareca penelope* (L.); *Mareca strepera* (L.)	Subspherical or ovoidal	8 × 6–8	–	–	–	Absent	Svanbaev & Rakhmatullina, 1967
	*Anas superciliosa* Gmelin	Subspheroidal to ellipsoidal	8–10 × 7–8 (8.8 × 7.7)	1.1–1.2 (1.1)	Barely or not discernible	Absent	Present, large, dense granules diffused	Present study
*Eimeria nyroca* Svanbaev & Rakhmatullina, 1967	*Aythya nyroca* (Güldenstädt)	Ovoidal	11–13 × 8–11	–	–	–	Present, granular	Svanbaev & Rakhmatullina, 1967
*Eimeria saitamae* Inoue, 1967	*A. platyrhynchos*	–	–	–	–	–	–	Inoue (1967)
*Eimeria schachdagica* Musaev, Surkova, Jelchiev & Alieva, 1966	*A. platyrhynchos*	Ovoidal	(9.2 × 8.4)	–	–	–	Present, small, granular	Musaev et al. (1966)
*Eimeria somateriae* Christiansen, 1952	*Clangula hyemalis* (L.)	–	(11 × 6)	–	–	–	Absent	Christiansen (1952)

*Note*: *E. boschadis* and *E. somateriae* are kidney parasites, while the remaining species are intestinal parasites.

a Range (Mean).

Morphologically, *E. anatis* is easily confused with *W. philiplevinei* due to the difficulty of distinguishing the sporocyst residuum and the number of sporozoites in their sporocysts. In this context, Duszynski et al. (2000) considered that the descriptions, photomicrographs and line drawings of *Wenyonella* spp. were inadequate; additionally, many species have been described and named from degenerate oöcysts. Thus, Duszynski et al. (2000) suggested that all species identified as *Wenyonella* should be viewed dubiously and considered *species inquiriendae*. Specifically for *W. philiplevinei*, Duszynski et al. (2000) considered that both the line drawing and photomicrograph suggest that the refractile bodies and/or sporozoites were all confused in the original description of Leibovitz (1968) and other studies reviewed by Gajadhar et al. (1983). In the oöcysts identified as *E. anatis* in the present study, two sporozoites were clearly observed with their anterior and posterior refractile bodies in each sporocyst, justifying that the material belongs to the genus *Eimeria*. Furthermore, the results of the phylogenetic analysis including the newly generated 18S sequence for *E. anatis* showed its inclusion into a clade of *Eimeria* spp. from ducks, while being distant from the only partial 18S sequence from oöcysts identified as *W. philiplevinei* by Wu et al. (2013).

*Tyzzeria perniciosa* is the type-species of the genus *Tyzzeria*, which consists of coccidia with oöcysts containing eight sporozoites without sporocysts. All consensually valid species are recorded from birds of the order Anseriformes. Descriptions in hosts of other vertebrate classes were published, although they must be misidentifications, such as *Tyzzeria boae* Lainson & Paperna, 1994 described from the red-tailed boa *Boa constrictor* L. and *Tyzzeria chalcides* Probert, Roberts & Wilson, 1988 described from the ocellated skink *Chalcides ocellatus* (Forskål), which potentially represent a species of *Klossiella* and a species of *Choleoeimeria*, respectively misidentified from oöcysts that sporulated abnormally (Duszynski et al., 1998).

The most frequently reported species in the literature are *T. perniciosa* from teals, mallards and other ducks (Anatinae) and *Tyzzeria parvula* (Kotlan, 1933) from geese (Anserinae) (Berto et al., 2007). Although the oöcysts of these species are morphologically very similar, they are specialised for parasitism at the subfamily level, i.e. *T. parvula* does not infect teals, mallards and ducks, just as *T. perniciosa* does not infect geese, even in experimental infections (Berto et al., 2007).

In addition to *T. perniciosa*, *Tyzzeria pellerdyi* Bhatia & Pande, 1966 was described and reported from *Anas* spp. in some studies in the 1960s, 1970s and 1980s. However, there is no morphological or biological differentiation that so far justifies and fundamentally defines *T. pellerdyi*. As this species was described after *T. perniciosa*, it is likely that *T. pellerdyi* is a junior synonym of *T. perniciosa* (Gajadhar et al., 1983; Duszynski et al., 1998) (Table 3). Similarly, two species, i.e. *Tyzzeria allenae* Chakravarty & Basu, 1946 and *Tyzzeria chenicusae* Ray & Sarkar, 1967, were described from the cotton pygmy-goose *Nettapus coromandelianus* (Gmelin); this host, in spite of the common name, belongs to the Anatinae. The oöcysts of these species were described with some morphometric differences that differentiate them from the original description of *T. perniciosa* and from the description provided here; however, these species have not been reported since their original descriptions (Table 3).

**Table 3:** Comparative morphological data for *Tyzzeria* spp. recorded from ducks (Anseriformes: Anatidae: Anatinae).

Species	Host	Oöcyst					Sporozoite				Reference
		Shape	Size (μm)^a^	Shape index	Residuum (μm)^a^	Wall (μm)^a^	Shape	Size (μm)^a^	Refractile body	Nucleus	
*Tyzzeria perniciosa* Allen, 1936	*Anas platyrhynchos* L.	Ellipsoidal	10–13 × 9–11	–	Present, large, composed of variously sized granules	Relatively thick	Curved, with one end more rounded and broader	(10.0 × 3.5)	–	–	Allen (1936)
	*Anas superciliosa* Gmelin	Ellipsoidal	10–11 × 7–8 (10.7 × 7.4)	1.4–1.5 (1.4)	Present, granules of different sizes usually clustered at one end of the oöcyst, *c.*2.5	Smooth, 0.4–0.7 (0.6)	Curved and tapered at anterior end	6–8 × 1–2 (6.9 × 1.8)	Robust, prominent	Not discernible	Present study
*Tyzzeria alleni* Chakravarty & Basu, 1946	*Nettapus coromandelianus* Gmelin	Ovoidal	14–17 × 10–12	–	Present, coarsely granular, *c.*6.4	–	Tapered at one end	5.3–6.5	–	Present, central	Chakravarty & Basu (1946)
*Tyzzeria pellerdyi* Bhatia & Pande, 1966	*Mareca strepera* (L.); *Aythya nyroca* (Güldenstädt); *Spatula clypeata* (L.); *Anas carolinensis* Gmelin; *A. platyrhynchos*	Subspherical to ovoidal	11–16 × 8–11 (13.0 × 10.0)	–	Present, *c.*4.0–5.0	Smooth, 0.5–0.7	Banana-shaped	(8.5 × 2.0)	Prominent	Present, central	Bhatia & Pande (1966); Bristol et al. (1981)
*Tyzzeria chenicusae* Ray & Sarkar, 1967	*N. coromandelianus*	Broad and cylindrical	20–28 × 14–20 (24.8 × 16.8)	(1.5)	Large, compact, at one pole of the oöcyst	(1.4)	Club-shaped	(13.2 × 4.2)	Present at the broader end	–	Ray & Sarkar (1967)

*Note*: All species are intestinal parasites.

a Range (Mean).

Our phylogenetic analyses provided strong evidence that the newly generated sequences from *E. anatis* in the Pacific black duck both at the 18S rRNA and *cox*1 loci were most close to those from domestic goose (GenBank: KP789171, KJ000077 and MT833388). As this is, to the best of our knowledge, the first study using molecular tools to the identification of duck coccidia, further similar studies on additional species of coccidia parasitic in ducks would be beneficial to the taxonomy of duck coccidia and assessment of their relationships with coccidian species parasitic in other host groups.

This study has revealed that, besides infecting the mallard *A. platyrhynchos*, *E. anatis* also infects the Pacific black duck. Hybridisation (interbreeding) between the introduced mallard and the Pacific black duck in Australia occurs at a rate of around 1.5% (Taysom, 2016), so it is likely that these two species of duck share some of their coccidian species as well.

## 5 Conclusion

In conclusion, the coccidia *E. anatis*, *E. aythyae*, *E. krylovi* and *T. perniciosa* are redescribed with supplementary morphological data, in order to ensure and facilitate their future identification from *A. superciliosa* or from other duck species. In addition, a genotypic characterization of *E. anatis* and taxonomic remarks on species and genera of dubious validity reported from Anseriformes are provided, aiming to contribute to the knowledge of coccidian species of ducks.

## CRediT author statement

Bruno P. Berto: morphological identification of the species, preparation of line drawings, writing - review & editing. Belinda Brice: coccidian primary screening and identification, writing - original draft and paper reviewing. Gwyneth Thomas: sample collection and coccidian primary screening, writing - review & editing. Aileen Elloit: oöcyst imaging, morphological identification of the species, writing - review & editing. Alireza Zahedi: oöcyst isolation, DNA extraction, PCR, sequencing, writing - review & editing. Rongchang Yang: overseeing and coordinating this study, phylogenetic analysis, writing - review & editing. All authors read and approved the final manuscript.

## Data availability

The newly generated sequences for *E. anatis* are deposited in the GenBank database under the accession numbers OL604501 (18S rDNA) and OL656104 (*cox*1). Photomicrographs and line drawings of the oöcysts are deposited and available (http://r1.ufrrj.br/labicoc/colecao.html) in the Parasitology Collection of the Laboratório de Biologia de Coccídios, at UFRRJ, under repository numbers 120/2021 (*E. anatis*), 121/2021 (*E. aythyae*), 122/2021 (*E. krylovi*) and 123/2021 (*T. perniciosa*), along with the photovouchers of the *A. superciliosa* specimen.

## Ethical approval

Not applicable.

## Funding

Official funding for this study was not available.

## Declaration of competing interests

The authors declare that they have no known competing financial interests or personal relationships that could have appeared to influence the work reported in this paper.
